# Natural frequency trees improve diagnostic efficiency in Bayesian reasoning

**DOI:** 10.1007/s10459-020-10025-8

**Published:** 2021-02-12

**Authors:** Karin Binder, Stefan Krauss, Ralf Schmidmaier, Leah T. Braun

**Affiliations:** 1 Mathematics Education, Faculty of Mathematics, University of Regensburg, Universitätsstraße 31, 93053 Regensburg, Germany; 2 Medizinische Klinik und Polklinik IV, Klinikum der Universität München, LMU Munich, Munich, Germany

**Keywords:** Bayesian reasoning, Clinical reasoning, Diagnostic efficiency, Natural frequencies, Undergraduate medical students

## Abstract

When physicians are asked to determine the positive predictive value from the a priori probability of a disease and the sensitivity and false positive rate of a medical test (Bayesian reasoning), it often comes to misjudgments with serious consequences. In daily clinical practice, however, it is not only important that doctors receive a tool with which they can *correctly* judge—the *speed* of these judgments is also a crucial factor. In this study, we analyzed accuracy and efficiency in medical Bayesian inferences. In an empirical study we varied information format (probabilities vs. natural frequencies) and visualization (text only vs. tree only) for four contexts. 111 medical students participated in this study by working on four Bayesian tasks with common medical problems. The correctness of their answers was coded and the time spent on task was recorded. The median time for a correct Bayesian inference is fastest in the version with a frequency tree (2:55 min) compared to the version with a probability tree (5:47 min) or to the text only versions based on natural frequencies (4:13 min) or probabilities (9:59 min).The score *diagnostic efficiency* (calculated by: median time divided by percentage of correct inferences) is best in the version with a frequency tree (4:53 min). Frequency trees allow more accurate *and* faster judgments. Improving correctness and efficiency in Bayesian tasks might help to decrease overdiagnosis in daily clinical practice, which on the one hand cause cost and on the other hand might endanger patients’ safety.

## Introduction

### Importance of Bayesian reasoning for medical students and physicians

In daily clinical practice, physicians are often confronted with so-called Bayesian reasoning situations: For example, when they have to explain test results of a mammogram, it is important for the patient to know what exactly these results mean, that means how likely it is that a positive result indicates a sickness. It is already known that physicians and also medical students are prone to errors at correctly estimating the probability of certain diseases and at interpreting test results (Eddy 1982; Hoffrage and Gigerenzer 1998; McDowell and Jacobs 2017).

We illustrate the situation in the breast cancer screening problem (adapted from Eddy 1982):

*Screening for breast cancer—Probability format:*

The probability of breast cancer is 1% for a woman of a particular age group who participates in a routine screening (a priori probability, also called prevalence P(B)). If a woman who participates in a routine screening has breast cancer, the probability is 80% that she will have a positive mammogram (sensitivity P(M + |B)). If a woman who participates in a routine screening does not have breast cancer, the probability is 9.6% that she will have a false-positive mammogram (false-alarm rate P(M + |¬B)).

What is the probability that a woman who participates in a routine screening and has a positive mammogram actually has breast cancer?

Most physicians in former studies assumed this probability to be between 70 and 80%, which is far from the correct positive predictive value (Eddy 1982; Hoffrage and Gigerenzer 1998). A wide variety of empirical studies have shown that physicians, medical staff, and patients (Garcia-Retamero and Hoffrage 2013; Hoffrage and Gigerenzer 1998) have difficulties with problems of this kind. However—maybe due to this misjudgment—the mammography screening for breast cancer is heavily promoted in many countries as necessary for every woman in a particular age group although it is very expensive (Gigerenzer and Gray 2011) and its medical benefit can be questioned seriously (Wegwarth and Gigerenzer 2013).

The *a posteriori probability* P(B|M+), which is the relevant one for patients, is also called the *positive predictive value* of a medical test. Given the prevalence of the disease P(B), the *sensitivity* of the test P(M + |B) and the *false-alarm rate* of the test P(M + |¬B), the positive predictive value can be calculated, for instance, with the help of the Bayes’ theorem. In the example above, the Bayes’ theorem shows that the actual probability of breast cancer given a positive mammogram P(B|M+) is only about 7.8%.

$$ \begin{aligned} P\left( {B|M + } \right) & = \frac{{P\left( {M + |B} \right)P\left( B \right)}}{{P\left( {M + |B} \right)P\left( B \right) + P\left( {M + |\neg B} \right)P\left( {\neg B} \right)}} \\ & = \frac{80\% \cdot 1\% }{{80\% \cdot 1\% + 9.6\% \cdot 99\% }} \\ & \approx 7.8\% \\ \end{aligned} $$

Fortunately, there are two effective strategies for overcoming occurring cognitive illusions and helping people to understand statistical information—namely, natural frequencies and visualizations (Binder et al. 2015; McDowell and Jacobs 2017; Spiegelhalter et al. 2011).

### Strategies to overcome Bayesian reasoning errors: Natural frequencies and visualizations

#### Natural frequencies

Rather than presenting all statistical information in Bayesian reasoning situations in the format of conditional probabilities and percentages, one can provide natural frequencies instead. In a seminal paper, Gigerenzer and Hoffrage (1995) translate the numbers in the breast cancer screening problem into natural frequencies in the following way:

*Screening for breast cancer—Natural frequency format:*

100 out of 10,000 women of a particular age group who participate in a routine screening have breast cancer. 80 out of 100 women who participate in a routine screening and have breast cancer will have a positive mammogram. 950 out of 9,900 women who participate in a routine screening and have no breast cancer will have a false-positive mammogram.

How many of the women who participate in a routine screening and receive positive mammograms have breast cancer?

It is now easier to understand that there are 80 + 950 women with positive mammograms in the sample, and that only 80 out of these 1,030 women actually have breast cancer, which results in a positive predictive value of about 7.8%. With the natural frequency version significantly more people are able to make the correct inference (Gigerenzer and Hoffrage 1995; McDowell and Jacobs 2017; Siegrist and Keller 2011; Weber et al. 2018). A current meta-analysis has shown that the effect of natural frequencies in Bayesian reasoning is quite robust; the typical performance for the probability versions of Bayesian reasoning tasks across studies is 4%, while it is 24% for the corresponding natural frequency versions (McDowell and Jacobs 2017). Interestingly, some of the participants fail to solve the tasks in natural frequency versions correctly, because they translate the given information back into complicated probabilities (Weber et al. 2018). Even though the natural frequency effect is known since about 25 years now, Bayesian reasoning problems are often explained to medical students with the help of probabilities (Kirkwood and Sterne 2010).

Besides changing information format from probabilities to natural frequencies there is a second strategy for improving Bayesian reasoning, namely, visualizations (Binder et al. 2015; Binder et al. 2018; Brase 2008; Khan et al. 2015; Spiegelhalter et al. 2011).

#### Visualizations

**Fig. 1:**
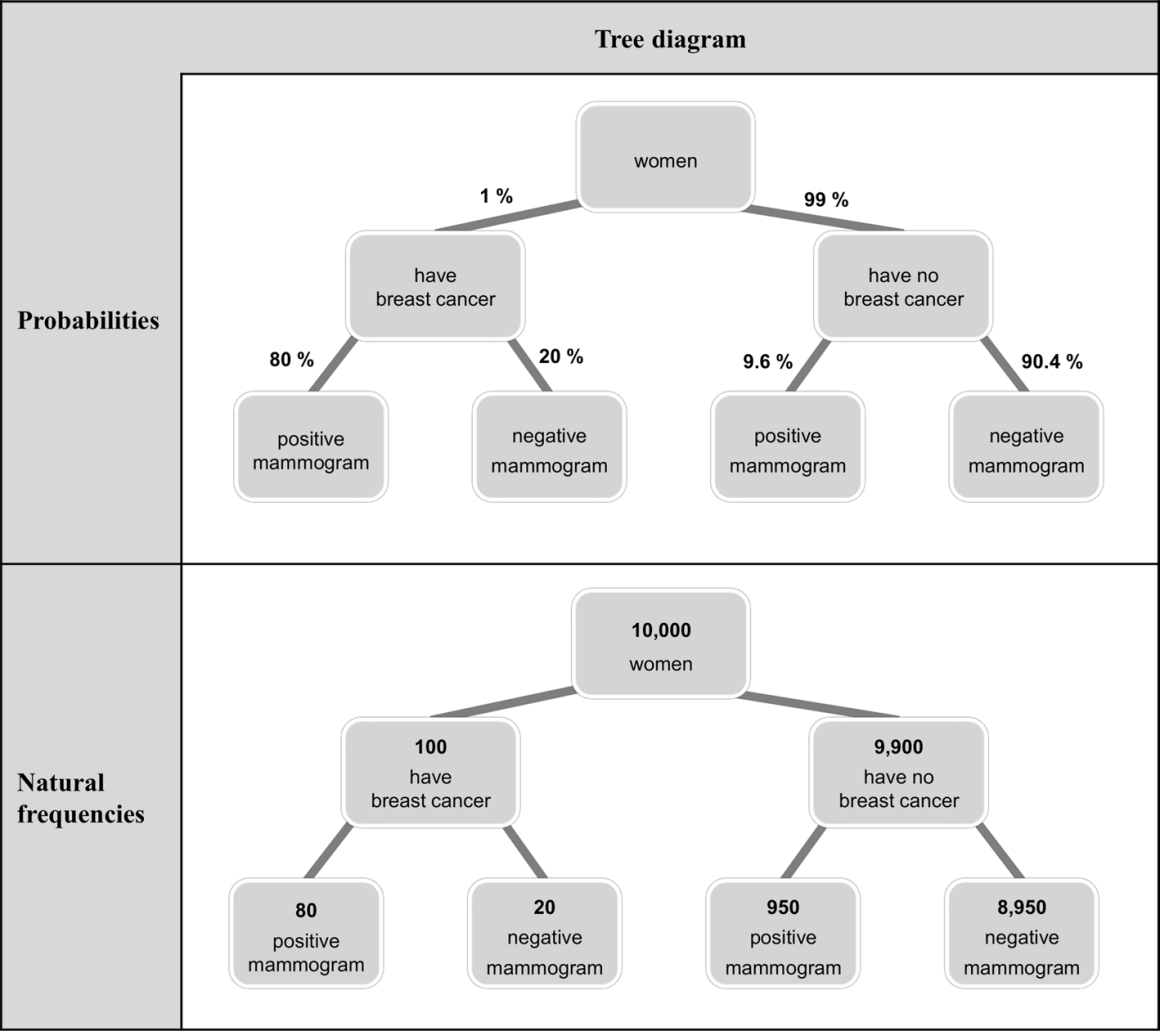
Tree diagram with probabilities (above) or natural frequencies (below) for the breast cancer screening problem

There are several different visualizations with respect to Bayesian reasoning tasks, like icon arrays (Brase 2008,2014; Galesic et al. 2009; Tubau et al. 2019; Zikmund-Fisher et al. 2014), 2 × 2-tables (Binder et al. 2015; Eichler et al. 2020; Steckelberg et al. 2004), tree diagrams (Binder et al. 2015; Budgett et al. 2016; Friederichs et al. 2014; Steckelberg et al. 2004; Yamagishi 2003), double-trees (Binder et al. 2020; Eichler et al. 2020), net diagrams (Binder et al. 2020), Euler diagrams (Micallef et al. 2012; Sirota et al. 2014), roulette-wheel diagrams (Brase 2014; Yamagishi 2003), frequency grids (Garcia-Retamero and Hoffrage 2013; Sedlmeier and Gigerenzer 2001), and unit squares (Böcherer-Linder and Eichler 2017; Pfannkuch and Budgett 2017). Most of these visualizations usually do not contain any numbers (e.g., icon arrays). However, tree diagrams usually contain numbers (for an exception see Friederichs et al. 2014) and can be filled with probabilities or natural frequencies (see Fig. 1). However, only tree diagrams containing frequencies in the nodes, not tree diagrams with probabilities at the branches or without any numerical information, significantly foster insight into Bayesian reasoning problems (Binder et al. 2015, 2018). All the mentioned visualizations have already been tested empirically (Eichler et al. 2020; Khan et al. 2015; Spiegelhalter et al. 2011). However, there is evidence that not all types of visualizations support people in their decision-making processes. Furthermore, whereas most of the discussed visualizations entails an enormous effort to be produced (e.g., by paper & pencil), tree diagrams can be depicted very quickly (Binder et al. 2015, 2018). As a result, the tree diagram can also be used very well for the training of medical students and physicians.

Because so far *speed of Bayesian inferences* has rarely been investigates so far, we will shed light on this question in the following.

### Time on task and diagnostic efficiency

As mentioned before, physicians are confronted with Bayesian reasoning situations quite often in their daily clinical practice. To treat patients correctly and to advise them wisely—for example how to deal with a positive mammogram—it is important that physicians are taught in Bayesian reasoning. Besides making correct decisions, in daily clinical practice it is also important to make correct decisions fast: For example, the average time a physician has to treat a patient in a family practice is about 5 min (Falk Osterloh 2012). So, although it might be desirable to invest as much time as needed in a certain patient, this wish simply does not reflect real working conditions. It is already known, that diagnostic efficiency, which can be defined as the number of correctly solved clinical cases divided by the time needed for diagnosis is a competence facet of physicians (Braun et al. 2017) that can be improved by scaffolding such as representation prompts. Furthermore, also other instructional approaches such as structured reflection and feedback can reduce the time needed for diagnosing (Braun et al. 2019). In sum, instructions can help medical students to solve diagnostic problems faster without being less accurate.

There are, however, only few studies that deal with the speed of diagnosis in Bayesian tasks: On the one hand, an eye tracking study, which only looked at tasks with natural frequencies, indicates that incorrect Bayesian judgements are given more quickly than correct judgements (Reani et al. 2018). On the other hand, another eye tracking study found no clear difference in the processing time between correct and incorrect answers (Bruckmaier et al. 2019). Furthermore, it seems that visualizations—and especially tree diagrams—increase the diagnostic speed of Bayesian tasks formulated in natural frequencies (Reani et al. 2018).

Previous studies that have already dealt with the time required to complete a Bayesian task, for example, have shown that the processing time is increased with the number of mental steps required (e.g., when the question sample is not compatible in size to the target sample of the question, participants took longer to answer the Bayesian reasoning task; Ayal and Bayth Marom 2014).

In the present study, we will address the influence of information format (probabilities vs. natural frequencies) and visualization (text only vs. tree only) on processing speed systematically for the first time. In this study the following research question was ought to be answered (besides a replication of accuracy effects): Do *natural frequencies* and *tree diagrams* help to reach a diagnosis faster in Bayesian reasoning tasks? We hypothesized that the combination of natural frequencies and tree diagrams is the best combination not only to foster accuracy, but also diagnostic efficiency.

## Material and methods

### Participants

111 medical students (66 female, 44 male, 1 unknown) have participated in this study in July and August 2018 and proceeded all cases. Participants were on average 24.61 years old (*SD* = 7.97), the average year of medical education was *M* = 6.95 (*SD* = 3.46), the school leaving examination grade (1 = best grade to 6 = worst grade) was *M* = 1.50 (*SD* = 0.54) and the grade of preliminary medical examination was *M* = 3.24 (oral, *SD* = 1.39) and *M* = 3.34 (written, *SD* = 1.34). They had on average 1.98 weeks (*SD* = 1.73) of clinical experience.

All students were informed that their participation was voluntary, and anonymity was guaranteed. Participants had given their prior written consent to participating in the study. For participating, they received a financial incentive of ten Euros. The study was approved by the Ethical Committee of the Medical Faculty of LMU Munich (Number: 17-829).

### Study design

**Fig. 2:**
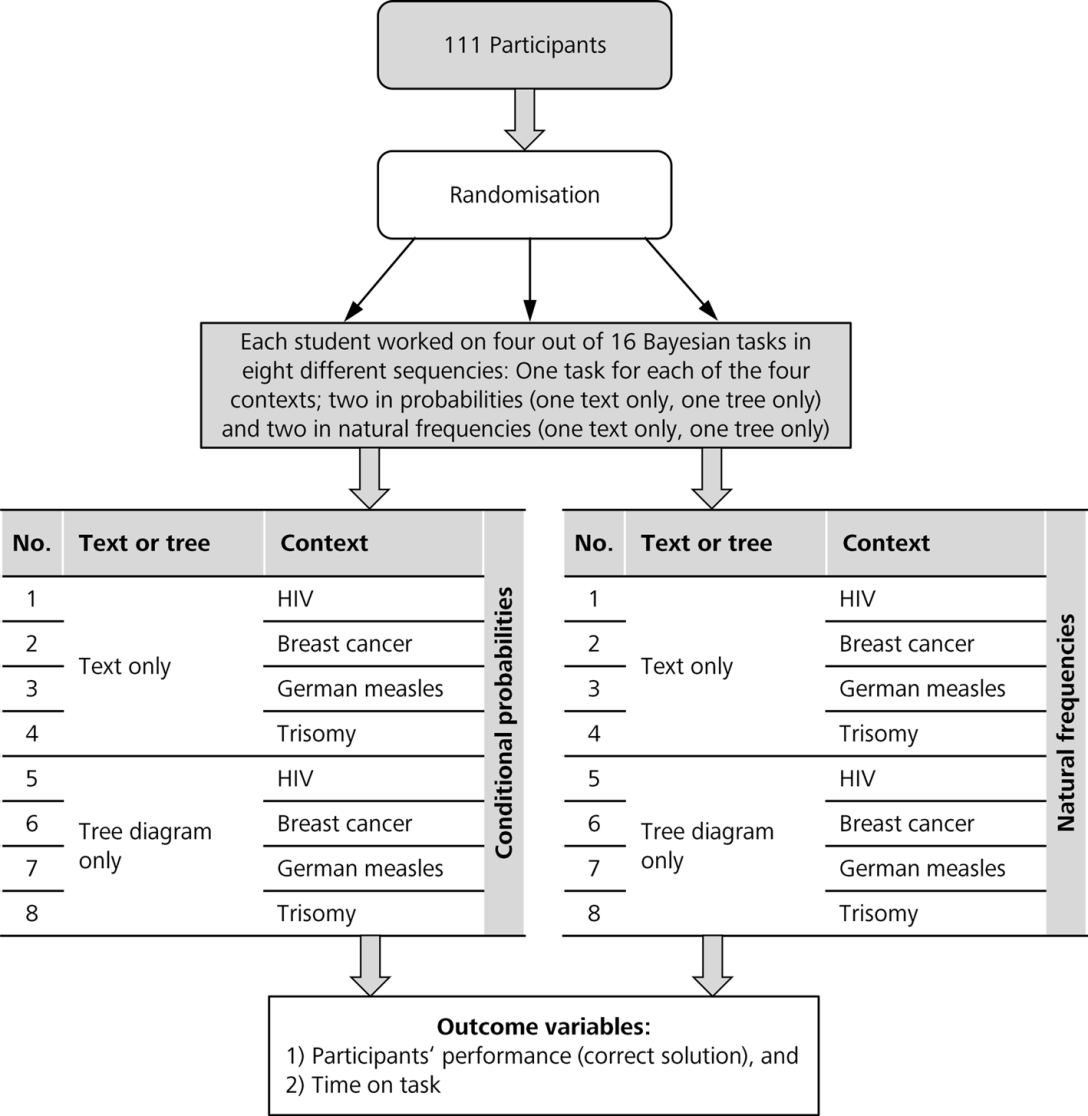
Study design

Since both purely textual formulations and tree diagrams per se principally contain all numbers that are needed for Bayesian inferences, we decided to split both representations and present either one of them to participants. Figure 2 shows the 2(information format: probabilities vs. natural frequencies) × 2(visualization: text only vs. tree only) × 4(contexts: not a factor of interest)-design of the prospective study with a quasi-experimental design. After a short introduction text, participants completed an electronic questionnaire regarding socio-demographic characteristics. Then, they worked within the electronic case simulation platform CASUS (Fischer et al. 1999) in a laboratory setting on four Bayesian situations (see Table 1 and “Appendix”). Overall time or time to spend on each case was not limited. Pocket calculators were distributed and students were allowed to use them at any point during the test.

### Material and medical encounters

Each participant worked on four different cases with the following medical encounters: HIV, breast cancer, Rubella and Trisomy (Eddy 1982; Gigerenzer and Hoffrage 1995; Prinz et al. 2015; Steckelberg et al. 2004). Each of these problems was realized by four versions: probabilities (text only), probability tree, natural frequencies (text only) and frequency tree. The problem formulation of the breast cancer screening problem can be found in Table 1 (the other problem formulations are depicted in the "Appendix").

**Table 1:** Problem formulations for the breast cancer screening contexts

	Breast cancer screening problem	
	Probability version	Natural frequency version
Medical situation	Imagine that you are a physician in a mammography screening center where women without symptoms are screened with mammograms for breast cancer	
	At the moment, you are advising a woman who has no symptoms but who has received a positive result from her mammogram. This woman wants to know what this result mean for her	
	For your answer, there is the following information available, which is bases on a random sample of women who have all undergone a mammography	
Presentation of information	•Text only	•Text only
	•Tree diagram only	•Tree diagram only
Text	The probability of breast cancer is 1% for a woman of a particular age group who participates in a routine screening. If a woman who participates in a routine screening has breast cancer, the probability is 80% that she will have a positive mammogram. If a woman who participates in a routine screening does not have breast cancer, the probability is 9.6% that she will have a false-positive mammogram	100 out of 10,000 women of a particular age group who participate in a routine screening have breast cancer. 80 out of 100 women who participate in a routine screening and have breast cancer will have a positive mammogram. 950 out of 9,900 women who participate in a routine screening and have no breast cancer will have a false-positive mammogram
Tree diagram	Probability tree (in the version with a tree diagram)	Frequency tree (in the version with a tree diagram)
Question	What is the probability that a woman with a positive mammogram actually has breast cancer?	How many of the women with a positive mammogram actually have breast cancer?
	Answer: _______	Answer: ____ out of _____

Each participant received two of the four problem contexts in probability format (one of them with a tree diagram the other one only with the text) and the other two problem contexts in natural frequency format (again one of them with a tree diagram the other one only with the text), with the order of context, information format and visualization varied systematically.

The overall time or the time on a certain task was not restricted, but students were informed that the time spent on the tasks was recorded (“Each task takes 5–10 min. In your daily work you have to make important decisions in limited time. Therefore, the processing time is not limited, but it is registered by the program.”).

### Coding

In accordance with Gigerenzer and Hoffrage (1995), a response elicited from a probability version was classified as correct if it was the exact Bayesian solution or rounded to the next whole percentage point above or below. In the natural frequency versions, responses were classified as correct only if both numbers (e.g., in the breast cancer screening solution of “80 out of 1,030”, both the 80 and the 1,030) were denoted correctly.

### Statistics

The sample size was derived by power analysis and thus based on effect sizes observed in previous studies using a similar design (Binder et al. 2018; McDowell and Jacobs 2017), which suggest a format effect close to 100% power (95% CI, 96.4% to 100%) with a sample size of about *N* = 120 students.

In order to statistically compare the effects of the information format and the types of visualization, we estimated (*generalized) linear mixed models* (with a logit link function) to predict 1) performance for the Bayesian reasoning task and 2) time for solving the task. In this model, we specified the probability version without a tree diagram as the reference category and included the possible explanatory factors “natural frequencies” and “tree diagram” via dummy coding.

## Results

### Participants’ performance (accuracy)

**Fig. 3:**
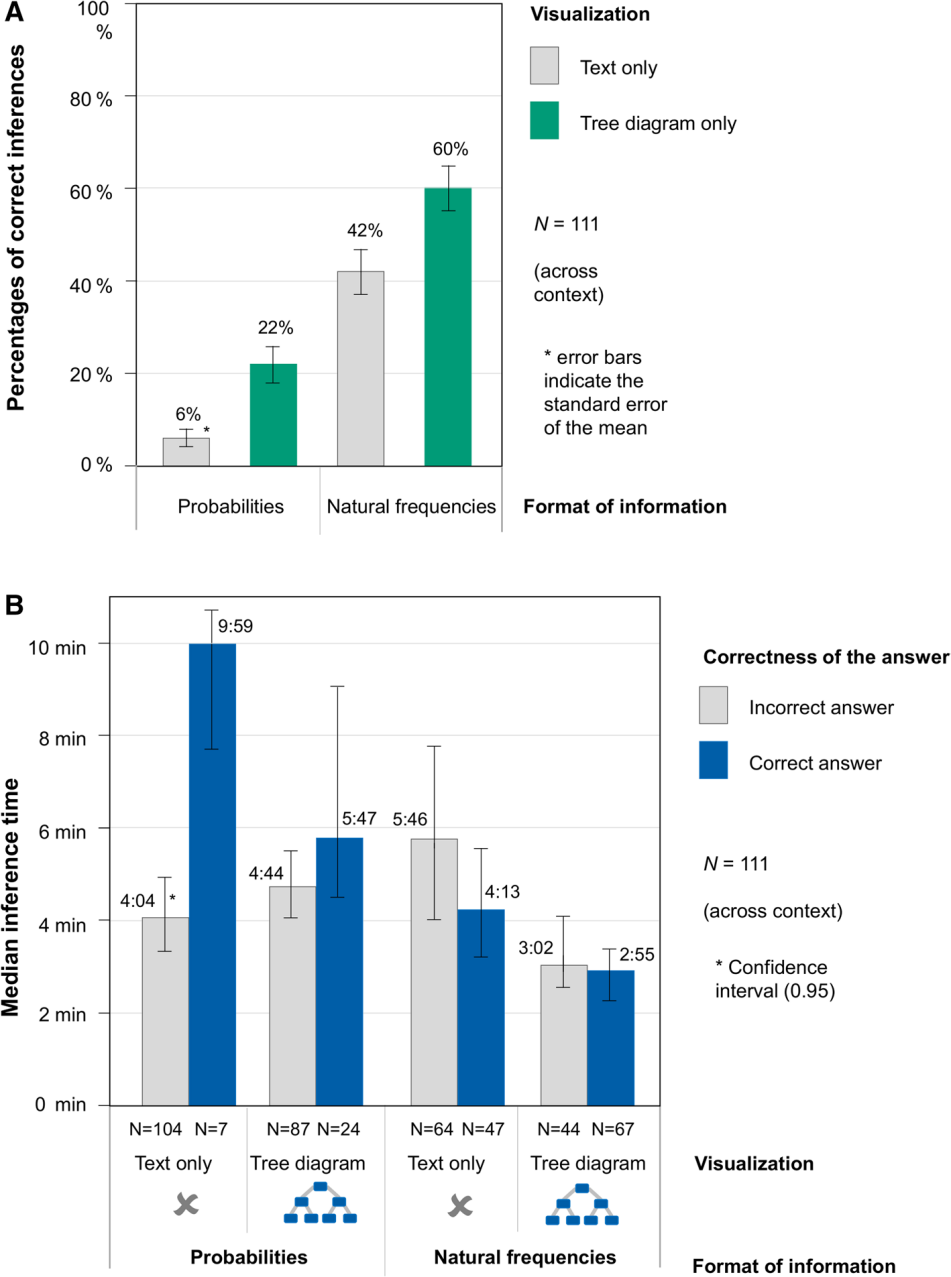
**a** participants’ performance in the Bayesian reasoning tasks (across contexts). **b**. Median time for solving one Bayesian reasoning tasks correctly or incorrectly (across contexts)

The probability versions of the Bayesian reasoning tasks were answered correctly by 6% (text only) or 22% (probability tree; see Fig. 3a, or Table 2, left). The performance rate in natural frequency versions was substantially higher; the rate was 42% (text only) and 60% (frequency tree). Thus, both natural frequencies and tree diagrams could increase the performance significantly.

**Table 2:** Overall results (across contexts): percentages of correct inferences, median, 1st and 3rd quartiles in seconds for the variable Speed and two different scores for the diagnostic efficiency

	Percentages of correct inferences	Median time for a diagnosis (1st Qrtl, 3rd Qrtl)	Median time for a correct diagnosis (1st Qrtl, 3rd Qrtl)	Score 1: Median time (all)/correct inferences	Score 2: Median time for a correct diagnoses /correct inferences
*Probabilities*					
Text only	6	4:36 min (2:24; 7:22)	9:59 min (9:15; 10:37)	1:16:40 h	2:46:26 h
Tree only	22	5:00 min (3:06; 8:29)	5:47 min (4:22; 9:24)	22:44 min	26:17 min
*Natural frequencies*					
Text only	42	4:36 min (3:01; 9:01)	4:13 min (2:51; 6:27)	10:57 min	10:02 min
Tree only	60	2:56 min (1:55; 6:18)	2:55 min (1:54; 3:58)	4:53 min	4:52 min

In a generalized linear mixed model (GLMM) for predicting the correctness of the answer, the (unstandardized) regression coefficients, both for natural frequencies (*b*_*1*_ = 2.96, *SE* = 0.39, *z* = 7.68, *p* < 0.001) and for presenting a tree diagram (*b*_*2*_ = 1.41, *SE* = 0.31, *z* = 4.60, *p* < 0.001), were significant (unstandardized regression coefficient: *b*_*0*_ = −3.58, *SE* = 0.46, *z* = −7.76, *p* < 0.001), indicating that both factors helps students in decision making (and therefore replicating previous effects). Other variables, however, such as the age, gender, grade of the participant, order of the task or context of the task (factor 3) had no significant influence on the performance in the task.

### Time for inference

In addition to the correctness of the answer, the speed of diagnosis is of great importance in everyday medical life. Table 2 shows the median time required by the medical students for each problem type, and also the median time for each problem type if one only considers the right answers. Furthermore, Fig. 3b shows the median time for each problem type, separated by correct and incorrect inferences. The median is reported instead of the arithmetic mean, since the times (as it is usually the case with processing times of participants) are distributed strongly right skewed.

Overall (and without taking correctness into account), the tasks are processed faster if they are shown with natural frequencies (*median* = 3:11 min, *CI*: [2:55; 3:32]) instead of probabilities (*median* = 7:41 min, *CI*: [5:04; 9:31], across text only and tree only versions). Furthermore, the tasks are processed faster, if they are shown with a tree diagram (*median* = 3:18 min, *CI*: [2:54; 4:03]) instead of a purely textual version (*median* = 4.36 min, *CI*: [3:20; 6:24], across probability and natural frequency versions). The effect of the tree diagram on diagnostic speed is particularly evident in the version with natural frequencies.

In a linear mixed model (LMM) for predicting the time to come to a Bayesian inference, the standardized regression coefficients, both for natural frequencies (*β*_*1*_ = −0.08, *SE* = 0.04, *t* = −2.10, *p* = 0.04) and for presenting a tree diagram (*β*_*2*_ = −0.16, *SE* = 0.04, *t* = −4.03, *p* < 0.001), were significant (unstandardized regression coefficient: *β*_*0*_ < 0.01, *SE* = 0.06, *t* < 0.01, *p* = 1.00). Other variables, however, such as the age, gender, grade of the participant or the order of the task or the context of the task had no significant influence on the time on solving the task.

Of particular interest, however, is the analysis of processing times for correct answers, as descriptively shown in Fig. 3b. In addition, we ran a further linear mixed model for predicting the time to come to a *correct* Bayesian inference. In this model, the standardized regression coefficients both for natural frequencies (*β*_*1*_ = −0.45, *SE* = 0.07, *t* = −6.77, *p* < 0.001) and for presenting a tree diagram (*β*_*2*_ = −0.34, *SE* = 0.06, *t* = −5.43, *p* < 0.001), were significant (unstandardized regression coefficient: *β*_*0*_ = 0.02, *SE* = 0.08, *t* = 0.21, *p* = 0.84).

### Efficiency in solving Bayesian reasoning tasks

Finally, it is possible to analyze accuracy and speed in combination. In Table 2 (right) two different possible scores regarding *diagnostic efficiency* are depicted. Score 1 divides the median time on task by the proportion of correct inferences. Lower values of this score indicate more correct and faster diagnoses. The best score occurs for the frequency tree (4:54 min), while the worst score occurs for the text only version with probabilities (1:16:40 h). A second possibility to calculate a score is to divide only the median time for a *correct* diagnosis by the proportion of correct inferences. Score 1 can also be interpreted by imagining people solving Bayesian tasks one after the other in a fixed format (e.g., probabilities with a tree): The score indicates the average time it takes for the first correct diagnosis to be given. For example, for answering the first version with a frequency tree correctly, it needs 4:53 min. On the other hand, it takes 1:16:40 h to answer the first probability task without a tree diagram correctly (see also Table 2).

## Discussion

This is the first study investigating the influence of information format (probabilities vs. natural frequencies) and tree diagrams (text only vs. tree only) on the efficiency in Bayesian reasoning. In sum, natural frequencies and tree diagrams can help medical students not only to answer these tasks more often accurate, but also more efficient. These results should affect medical education directly: Bayesian tasks should be taught by using frequency trees: it takes less time to answer these tasks correctly and—as the format is easier to understand for students—furthermore, the strategy is also more memorable than the formula of Bayes (Sedlmeier and Gigerenzer 2001). Therefore, it combines two advantages: Medical students can be easily prepared to solve Bayesian tasks correctly and they will be more efficient in their daily clinical practice.

Although is has been known for a long time that probabilities are not the best way to teach students Bayesian reasoning, it is still the standard teaching method in the curriculum of our medical school—and most likely in many other medical schools as well. This study shows that teaching Bayesian reasoning with natural frequencies and tree diagrams improves accuracy and efficiency of medical students. On this basis, medical courses should be revised to improve medical education and also to create changes in everyday clinical practice.

## Limitations and outlook

Although this study investigated diagnostic efficiency in Bayesian tasks quite comprehensively, a few limitations remain. First, we did not test any long term effects and whether medical students will use natural frequencies on their own (for example, whether they convert probabilities into natural frequencies when they are confronted with Bayesian-tasks; Weber et al. 2018). Furthermore, we did not compare different visualizations and cannot comment on the effect of other diagrams (e.g., net diagrams or double trees; Binder et al. 2020). These questions could be addressed in further studies.

Furthermore, besides pure calculation in Bayesian reasoning situations, it is also important that medical students learn, how to extract and asses evidence from scientific articles (Keller et al. 2017). In addition, using frequency trees to explain test results to patients might be a promising tool for doctor-patient communication and should be tested.

Although the risk literacy itself seems not to be dependent on the context, nevertheless, further studies might investigate the influence of the specific medical profession on the accuracy in Bayesian reasoning. For example, a gynecologist who is often confronted with the breast cancer screening problem in his daily clinical practice, might be better in solving those tasks as he is familiar with the correct solution.

Furthermore, the application of the Bayesian reasoning model has limitations in everyday clinical practice. For a lot of clinical signs, symptoms or even diagnostic tests, prevalence data or sensitivity and specificity are not defined or unknown (Moons et al. 1997). Therefore, the suggested strategy of using natural frequency trees is limited to Bayesian reasoning situations, where information on prevalence, sensitivity and specificity is known, and cannot be used in every possible scenario (in those cases specific heuristics might be helpful; Leuders and Loibl 2020).

As a consequence of our results (and former results on the natural frequency effect), we suggest a revision of the clinical training not only for medical students, but for physicians and other health careers such as nurses and physiotherapists as well. All health careers are confronted with Bayesian problems in their everyday clinical life but there do not seem to be adequate, systematic training concepts for all groups. Using tree diagrams with natural frequencies might be a first step for implementation (Kurz-Milcke et al. 2008).

## Conclusion

In this study with 111 participants, natural frequencies and frequency trees help medical students not only to answer Bayesian tasks more accurate, but also faster. In analyzing *diagnostic efficiency* one must distinguish between correct and incorrect inferences.

## Data Availability

The data of this study will be made publicly available after acceptance of the manuscript.

## Appendix

**Table:**

	Trisomy	
	Probability version	Natural frequency version
Medical situation	Imagine being a gynecologist in your own practice. For each pregnant woman, you perform a triple test for prenatal diagnosis between the 15th and 18th week of pregnancy in order to detect a possible trisomy 21 in the unborn child	
	You are currently counseling a pregnant woman who has received a positive test result in the triple test. This woman wants to know what this means for her unborn child	
	For your answer, only the following information is available, based on a sample of pregnant women who have also undergone a triple test	
Presentation of information	Text only	Text only
	Tree diagram only	Tree diagram only
Text	The probability that the unborn child has trisomy 21 is 0.18%	12 out of 6,760 unborn children have trisomy 21
	The likelihood that a pregnant woman will receive a positive triple test when the unborn child has trisomy 21 is 75%	In 9 of 12 unborn children with trisomy 21, the pregnant woman receives a positive triple test
	The likelihood that a pregnant woman will receive a positive triple test by mistake even though the unborn child does not have trisomy 21 is 5.9%	In 395 out of 6,748 unborn children without trisomy 21, the pregnant woman is mistakenly receiving a positive triple test
Tree diagram	Probability tree (in the version with a tree diagram)	Frequency tree (in the version with a tree diagram)
Question	What is the probability that her unborn child will actually have Trisomy 21?	How many unborn children with a positive triple test actually have trisomy 21?
	Answer: _________	Answer: _____ out of ______

**Table:**

	Rubella	
	Probability version	Natural frequency version
Medical situation	In the context of the German Pregnancy Accompanying Examination, a test for rubella infection of the expectant mother is mandatory, since rubella can lead to serious damage to the embryo	
	You are currently counseling a woman who has had rubella during her pregnancy. This woman wants to know what this means for her unborn child	
	For your answer, only the following information is available:	
Presentation of information	Text only	Text only
	Tree diagram only	Tree diagram only
Text	The probability that a child is born with damages that can be attributed to a disease of the mother is 0.5%	100 out of every 20,000 children are born with damage that can be attributed to a disease of the mother
	If a child with such damage is born, then the likelihood of the mother suffering from rubella during pregnancy is 40%	In 40 out of every 100 children born with such damage, the mother was diagnosed with rubella during pregnancy
	If a healthy child is born, then the likelihood of the mother suffering from rubella during pregnancy is 1%	In 199 out of 19,900 children born healthy, the mother had been suffering from rubella during pregnancy
Tree diagram	Probability tree (in the version with a tree diagram)	Frequency tree (in the version with a tree diagram)
Question	What is the likelihood that this mother will give birth to a child with damages that can be attributed to her mother's illness?	In how many of the women who have had rubella during pregnancy, is the child born with fetal damage?
	Answer: _______	Answer: ____ out of _____

**Table:**

	HIV	
	Probability version	Natural frequency version
Medical situation	Imagine being a doctor at an AIDS counseling center. In addition to individual counseling sessions, HIV tests are also carried out in this AIDS counseling center. For this purpose, a blood sample is taken from the client and an HIV test is carried out	
	They are currently counseling a low-risk client who has received a positive HIV test result. This client wants to know what this means for him	
	For your answer, you will only have the following information available, based on a sample of low risk individuals who have all been HIV tested:	
Presentation of information	Text only	Text only
	Tree diagram only	Tree diagram only
Text	The probability of a low-risk person being HIV-infected is 0.01%	100 out of every 1,000,000 low-risk individuals are HIV-infected
	The probability that a person will receive a positive HIV test result when infected with HIV is 99.7%	100 out of 100 people who are infected with HIV receive a positive result in the HIV test
	The likelihood of a person being mistakenly positive for the HIV test while not HIV-infected is 0.0004%	4 out of 999,900 people who are not infected with HIV mistakenly receive a positive result from the ELISA test
Tree diagram	Probability tree (in the version with a tree diagram)	Frequency tree (in the version with a tree diagram)
Question	What is the probability that this person is actually HIV-infected?	How many of those positively tested for HIV are actually HIV-infected?
	Answer: _______	Answer: ____ out of _____
